# The DNA repair enzyme, aprataxin, plays a role in innate immune signaling

**DOI:** 10.3389/fnagi.2023.1290681

**Published:** 2023-12-15

**Authors:** Helena B. Madsen, Louise I. Pease, Rebekah-Louise Scanlan, Mansour Akbari, Lene J. Rasmussen, Daryl P. Shanley, Vilhelm A. Bohr

**Affiliations:** ^1^Center for Healthy Aging, Institute of Cellular and Molecular Medicine, University of Copenhagen, Copenhagen, Denmark; ^2^CAMPUS for Ageing and Vitality, Newcastle University, Newcastle, United Kingdom

**Keywords:** APTX, innate immunity, DNA repair, ataxia, microglia, DNA- and RNA-sensing pathways, neurodegenerative diseases

## Abstract

Ataxia with oculomotor apraxia type 1 (AOA1) is a progressive neurodegenerative disorder characterized by a gradual loss of coordination of hand movements, speech, and eye movements. AOA1 is caused by an inactivation mutation in the *APTX* gene. APTX resolves abortive DNA ligation intermediates. APTX deficiency may lead to the accumulation of 5’-AMP termini, especially in the mitochondrial genome. The consequences of APTX deficiency includes impaired mitochondrial function, increased DNA single-strand breaks, elevated reactive oxygen species production, and altered mitochondrial morphology. All of these processes can cause misplacement of nuclear and mitochondrial DNA, which can activate innate immune sensors to elicit an inflammatory response. This study explores the impact of APTX knockout in microglial cells, the immune cells of the brain. RNA-seq analysis revealed significant differences in the transcriptomes of wild-type and APTX knockout cells, especially in response to viral infections and innate immune pathways. Specifically, genes and proteins involved in the cGAS-STING and RIG-I/MAVS pathways were downregulated in APTX knockout cells, which suggests an impaired immune response to cytosolic DNA and RNA. The clinical relevance of these findings was supported by analyzing publicly available RNA-seq data from AOA1 patient cell lines. Comparisons between APTX-deficient patient cells and healthy control cells also revealed altered immune responses and dysregulated DNA- and RNA-sensing pathways in the patient cells. Overall, this study highlights the critical role of APTX in regulating innate immunity, particularly in DNA- and RNA-sensing pathways. Our findings contribute to a better understanding of the underlying molecular mechanisms of AOA1 pathology and highlights potential therapeutic targets for this disease.

## 1 Introduction

**Figure 1:**
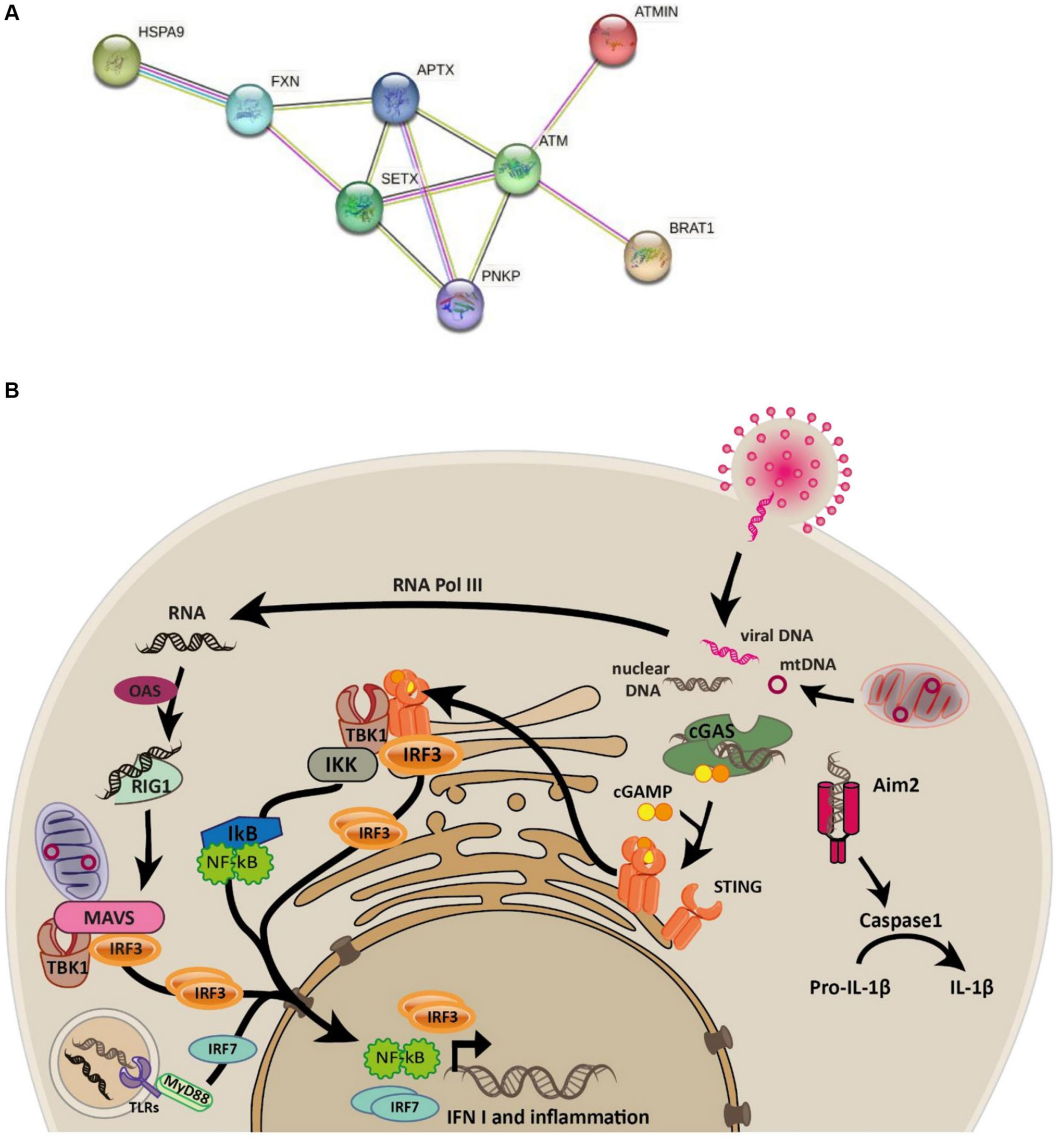
Connectivity between ataxia-associated genes and overview of the innate immune signalling pathways. **(A)** STRING network of proteins mutated in ataxias connected by edges colored by known associations (black = coexpression, pink = experimentally determined, purple = homologous proteins, light green = text mining, light blue = curated databases). **(B)** Graphical overview of the cGAS and RIG-I-MAVS signaling pathways elicited in response to cytosolic DNA and RNA that are known to trigger the phosphorylation and nuclear localization of IRF3 which subsequently transcribes interferon genes such as IFN1.

Ataxias are a spectrum of heterogenous progressive diseases where neurodegeneration is a common feature. Ataxias are characterized by slow progressive loss of coordination of hands, speech and eye movements, atrophy of the cerebellum (Moreira et al., 2001; Coutinho et al., 2023), and are associated with a range of phenotypes and mutations (Table 1). Biochemical and genetic tests, as well as family history are used for diagnosis (Klockgether, 2007). There are four subtypes of ataxia with oculomotor apraxia (AOA): AOA1 [mutations in APTX (Moreira et al., 2001) and/or PNKP (Bras et al., 2015)]; AOA2 [mutations in SETX (Airoldi et al., 2010)]; AOA3 [mutations in PIK3R5 (Tassan et al., 2012)]; AOA4 [mutations in PNKP (Paucar et al., 2016; Rudenskaya et al., 2019)]. All are associated with early disease onset and elevated alpha-fetoprotein, while AOA1, AOA2, and AOA4 are further associated with elevated cholesterol and hypoalbuminemia (Papadimitriou et al., 1996; Moreira et al., 2001; Sano et al., 2004; Quinzii et al., 2005; Kijas et al., 2006; Tada et al., 2010; Bogeski et al., 2011; Bras et al., 2015; Paucar et al., 2019; Kato et al., 2021; Coutinho et al., 2023). Conditions sharing phenotypic similarities could have related molecular causes and relationships between mutated genes. The STRING protein database (Szklarczyk et al., 2021) confirms this relationship for genes mutated in AOAs, ataxia telangiectasia (AT), and Friedereichs ataxia (Figure 1A). Many of the identified disease-causing genes (FXN, SETX, APTX, ATM and PNKP) are co-expressed (black lines), and experimentally determined relationships (pink lines) exist between APTX and PNKP, whose proteins are homologous (purple lines) as are SETX and ATM, SETX and FXN.

**Table 1:** Overview of some ataxias, genetic causes, clinical, and molecular association.

Ataxia	Abbreviations	Age of onset	Phenotype	Mutated genes
Ataxia with oculomotor apraxia Type I	AOA1	4	Elevated alpha-fetoprotein, hypoalbuminemia, decreased muscle Co-enzyme Q10, hypocalcemia, elevated total cholesterol, immunodeficiencies, sensitivity to single strand DNA breaks	APTX, PNKP (Bras et al., 2015; Moreira et al., 2001)
Ataxia with oculomotor apraxia Type II	AOA2, SCAR1	4.3	Elevated alpha-fetoprotein, hypoalbuminemia, decreased muscle Co-enzyme Q10, hypocalcemia, sensitivity to single-strand DNA breaks, immunodeficiencies	SETX (Airoldi et al., 2010)
Ataxia with oculomotor apraxia Type III	AOA3	<10	Elevated alpha-fetoprotein	PIK3R5 (Tassan et al., 2012)
Ataxia with oculomotor apraxia Type IV	AOA4	4.3	Elevated alpha-fetoprotein, elevated cholesterol, hypoalbuminemia	PNKP (Paucar et al., 2016; Rudenskaya et al., 2019)
Early onset ataxia with oculomotor apraxia	EAOH	4	Hypoalbuminemia, elevated total cholesterol, elevated alpha-fetoprotein	APTX (Kato et al., 2021)
Ataxia-telangiectasia	AT	3	Radiosensitivity, immunodeficiencies, elevated alpha-fetoprotein	ATM (van Os et al., 2017)
Spinocerebellar ataxia	OPCA1, OPCA4, CPD1, OPCA 616795	35	Elongated polyglutamine, coeliac disease	ATXN1,SCA1,SCA2,SCA3,SCA6,SCA7,SCA17 (Jacobi et al., 2013)
Friedereichs ataxia	FRDA, FRDA1, FA, FARR 229300	10	Abnormal lipid metabolism, diabetes, reduced mitochondrial respiration, free radical production	FXN (Walker et al., 1980)

APTX resolves abortive DNA ligation intermediates by catalyzing the release of adenylate groups covalently linked to 5′ phosphate termini at single-strand nicks and gaps to produce 5′-phosphate termini that can be efficiently rejoined. It is estimated that in mice 1,000,000 nicks are produced per cell cycle (Ahel et al., 2006; Rass et al., 2007; Tumbale et al., 2014; Fusco et al., 2017). It has been suggested that one cause of AOA1 is the accumulation of unrepaired DNA-adenylates due to loss of APTX activity (Ahel et al., 2006; Tumbale et al., 2014). Interestingly, depletion of APTX renders the mitochondrial genome, but not the nuclear genome, susceptible to damage, although the mechanisms are not clear (Akbari et al., 2015). Biochemical experiments demonstrated that APTX deficient cells had more 5’-AMP-DNA in mitochondrial DNA than in nuclear extracts (Akbari et al., 2015). CRISPR-Cas9-mediated knockout (KO) of *APTX* in human osteosarcoma cell line U2OS disrupted mitochondrial morphology, mitochondrial networks, decreased mitochondrial membrane potential (MMP) and increased reactive oxygen species (ROS) production, leading to impaired mitophagy (Akbari et al., 2015), also seen in AOA1 patient cell lines (Zheng et al., 2019). Persistent nuclear DNA damage has been linked to activation of poly ADP-ribose polymerase (PARPs), nicotinamide adenine dinucleotide (NAD+) depletion and mitochondrial dysfunction, processes that are thought to be causative in neurodegenerative disorders (Fang et al., 2014; Hou et al., 2021). The order of events leading to mitochondrial dysfunction in APTX deficient cells are currently unclear. However, it is known that loss of function of APTX leads to decreased ATP synthesis, increased DNA damage (particularly in mitochondria), increased ROS production and alterations in mitochondrial morphology, all are functions which have profound effect on normal cell function, including the release of self-DNA into the cytosol (Akbari et al., 2021). The presence of self- and foreign DNA in the cytosol can lead to immune responses and inflammation, which are activated by DNA sensing pathways including cGAS-STING signaling (Gregg et al., 2019; Akbari et al., 2021).

Cyclic GMP-AMP synthase (cGAS) is the primary protein that detects cytosolic double-stranded DNA (dsDNA) to invoke Type I interferon response (Gregg et al., 2019; Pasmanter et al., 2023). At the core of the cGAS-STING pathway, cGAS senses dsDNA leading to synthesis of cycling GMP-AMP (cGAMP), which serves as a secondary messenger. cGAMP interacts with STING, ultimately resulting in innate immune activation (Cai et al., 2014; Sun and Hornung, 2022). Further to this, cGAMP can travel between adjacent cells via gap junctions to activate the innate immune response in neighboring cells in a STING-dependent manner, allowing cells to confer protection to infection even when the interferon production in the infected cells is blocked by viral antagonists (Cai et al., 2014). Endosomal nucleic acids are detected by a subset of Toll-like receptors (TLR3, TLR7, TLR8 and TLR9), whereas cytosolic RNA activates the family of RIG-I like receptors to produce type I interferons and cytokines (Cai et al., 2014). The immune responses elicited by cells are thus tailored to the type of nucleic acid and its specific location within the cell (Figure 1B).

APTX impairment has been linked to immunodeficiency and mitochondrial dysfunction. We have previously shown that especially mitochondrial DNA is rendered susceptible to damage upon APTX depletion (Akbari et al., 2015) resulting in mitochondrial stress, impaired mitophagy and altered mitochondrial morphology (Zheng et al., 2019). In recent years, the immune system is becoming increasingly recognized as a contributor to several neurological and neurodegenerative disorders (Zang et al., 2022). Meanwhile, mitochondria have emerged as central organelles in innate immune signaling (Bahat et al., 2021), leading us to explore this link further in APTX deficiency. Thus, we knocked out the *APTX* gene in microglial cells, the immune cells of the brain, and stimulated the immune responses in WT and KO cell lines with dsDNA and Poly(I:C), an RNA mimic. RNA was harvested and sequenced after dsDNA stimulation, and key proteins were selected for further analysis at different stimulation timepoints, based on significant changes identified in the transcript analysis. Results were considered in conjunction with existing publicly available RNA-seq data for APTX loss of function (LOF) and healthy patient cell lines (E-GEOD-124412).

We first analyzed RNA-seq data to determine the effect of APTX KO in microglial cells, and whether these transcriptomic changes are reflective of the phenotypes seen in AOA. We found that APTX KO gives rise to altered viral responses, including DNA- and RNA-sensing innate immune pathways. Defective DNA- and RNA-sensing pathways were verified at the protein level by immunoblotting and fluorescence microscopy. Datasets from APTX LOF patient-derived cells also indicate similar dysregulation of viral responses, DNA- and RNA-sensing and the overall innate immune response.

These findings contribute to our understanding of APTX function beyond its direct role in DNA repair, and aids in the development of potential new targets to benefit AOA1 patients.

## 2 Results

### 2.1 APTX knockout results in altered innate immune responses and cell survival

**Figure 2:**
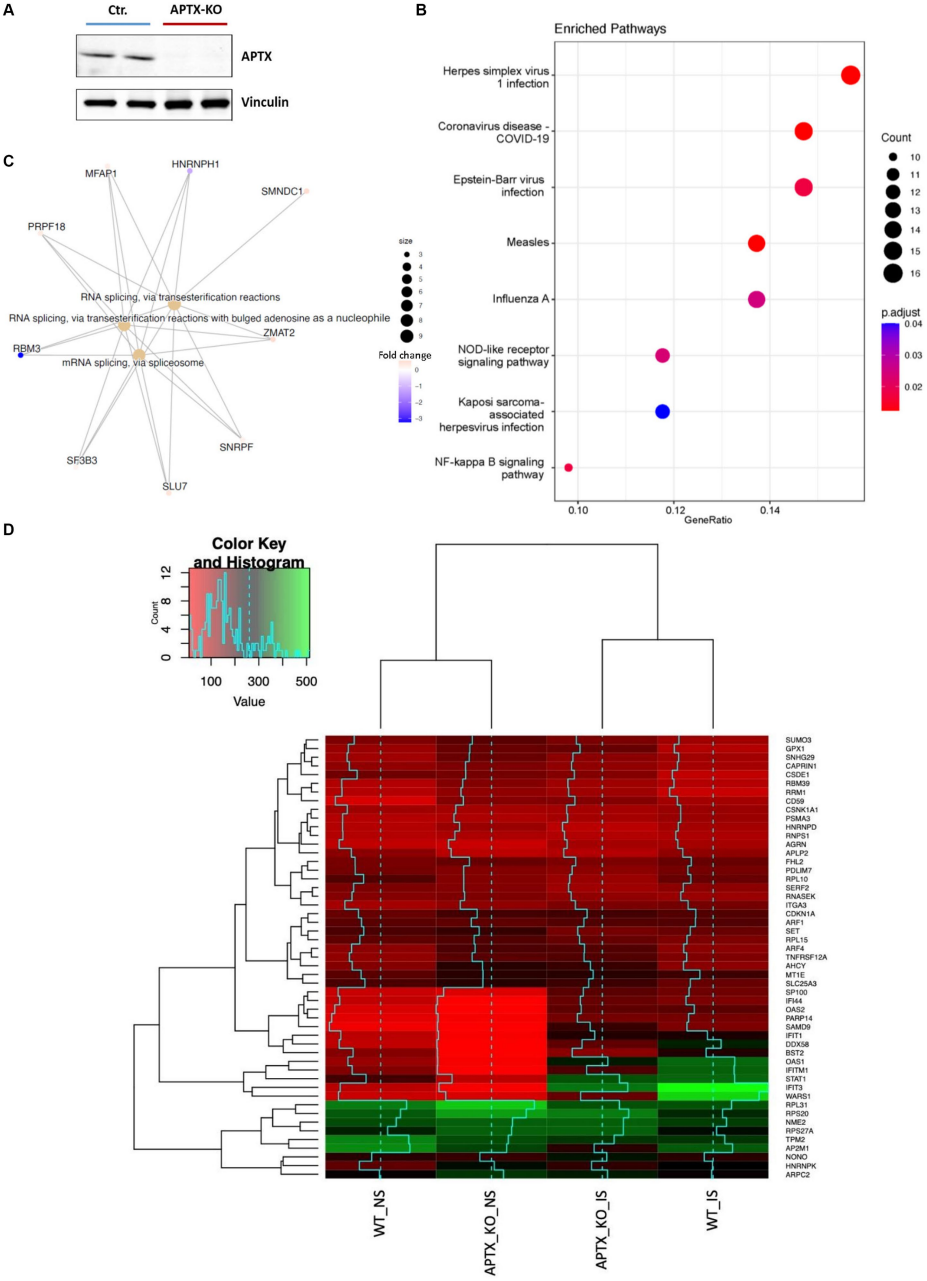
Verification of APTX KO and its transcriptional consequences. **(A)** Western blot image of APTX protein in control (Ctr) and APTX-KO microglial cells with vinculin as a control. Functional enrichment plots for network enriched genes identified as significantly (*q* < 0.05) differentially expressed genes belonging to network modules in WT vs. APTX KO microglial cell comparisons where **(B)** shows a dot-plot of enriched pathways and **(C)** shows conserved non-coding elements. **(D)** Hierarchically clustered heatmap of significantly (*q* < 0.05) differentially expressed genes with the high fold changes in expression in WT and KO cell lines with immune stimulation (IS) and without (NS) immune stimulation.

An APTX KO microglial cell line was generated using CRISP-Cas9 technique (Figure 2A). RNA-seq data was collected in triplicate from WT and APTX KO microglial cells and analyzed. Significantly (*q <* 0.05) differentially expressed (DE) genes were identified in WT and KO cells with (immune stimulated, IS) or without (not stimulated, NS) dsDNA exposure (Table 2). When WT cell lines are compared to KO mutants with and without IS, over 6,000 genes are DE (Table 2), highlighting that the transcriptomes of WT and APTX KO cell lines are fundamentally different. Further analysis was performed to investigate differences between transcripts, and it was found that more genes and transcripts were DE in KO cells than WT cells in non-stimulated and immune-stimulated samples (Supplementary Table S1). This could be due to the increased presence of nicked DNA in APTX KO cells, leading to decreased access to DNA and thus compromised accuracy of transcription.

**Table 2:** Summary of the number of significantly (*q* < 0.05) DE genes for each of the comparisons identified by RNA sequencing analysis.

Comparisons	Genes
WT_NS vs. WT_IS	6,837
WT_NS vs. APTX_KO_NS	6,748
WT_NS vs. APTX_KO_ IS	6,837
WT_IS vs. APTX_KO_IS	6,787
APTX_KO_NS vs. APTX_KO_IS	4,641

To explore the impact of APTX KO on the transcriptome, enrichment analysis was performed on DE genes. In NS cells, analysis found that many pathways enriched in WT compared to APTX KO were related to viral infections (such as measles, Epstein–Barr virus infection, and influenza A), but also to immune related pathways (NOD-like receptor signaling) and inflammatory pathways (NF-kB signaling pathway) (Figure 2B). Along with differences in response to viral infection, this analysis provides evidence that KO of APTX results in altered innate immunity as NOD-like receptor signaling has been described as a master regulator of innate immunity (Zhong et al., 2013). Differences in conserved non-coding elements (CNE) were also analyzed and it was found that the most enriched processes affected by an APTX KO is RNA splicing via transesterification and mRNA splicing via spliceosome (Figure 2C), suggesting that APTX KO results in altered splicing dynamics, which is supported by the high number of transcripts found (Supplementary Table S1). In addition to these enriched processes, nine transcripts were identified by CNE analysis, with HNRNPH1 and RBM3 identified as highly expressed in APTX KO cells compared to WT cells (Figure 2C). HNRNPH1 is an RNA binding protein which assembles on mRNA to regulate gene expression (Neckles et al., 2019), and RBM3 is a cold- and hypoxia-inducible protein (Danno et al., 1997), and expression is regarded as a potential cell survival mechanism (Zhou et al., 2017). RBM3 functions in cell cycle progression (Ehlén et al., 2010; Matsuda et al., 2011), and regulating protein synthesis through microRNA levels (Dresios et al., 2005). As RBM3 is more active in APTX KO cells, this suggests that APTX KO cells are in a stressed state with an altered cellular makeup, which can lead to immune defects. As both have functions relating to the regulation of gene expression, this further supports the altered splicing dynamics identified when APTX is knocked out (Figure 2C; Supplementary Table S1).

Expression levels of genes, which were significantly (*q* < 0.05) DE across all conditions, were visualized using hierarchically clustered heatmapping (Figure 2D), illustrating that although there are clear differences between WT and APTX KO cells, conditions cluster together based on stimulation status. Upon IS, both WT and APTX KO cell lines show increased expression of a cluster of co-expressed transcripts (SP1100, IFI44, OAS2, PARP14, SAMD9, IFIT1, DDX58, BST2, OAS1, IFITM1, STAT1, IFIT3, and WARS1). Of these transcripts IFITM1, IFIT3 and WARS1 have lower expression levels in APTX KO cell lines, all genes which are regulated by interferon activity.

Only four transcripts are significantly DE (*q <* 0.05) in response to IS in both cell lines (Table 3). These transcripts belong to three genes: IFIH1, TGFB2 and PRKCA, and are associated with three KEGG pathways all related to infection and infection detection (RIG-I-like receptor signaling pathway, Malaria, and African trypanosomiasis). Two transcripts for the IFIH1 gene were identified, showing that two isoforms of the gene were present in both WT and KO mutant cell lines. In some cases, different transcripts were present in the WT and KO cell lines after IS but were present in common KEGG pathways, with the majority of these related to viruses and infection (Supplementary Table S2).

**Table 3:** Summary of genes significantly (*q* < 0.05) increased in response to immune stimulation in both WT and APTX KO mutant.

Transcript name	Gene name	KEGG Term	Comparison	Fold Change	qVal
ENST00000649979	IFIH1	RIG-I-like receptor signaling pathway	APTX_KO_NS vs. APTX_KO_IS	0.09108	0.00531
ENST00000649426	IFIH1	RIG-I-like receptor signaling pathway	APTX_KO_NS vs. APTX_KO_IS	0.48754	0.00474
ENST00000366930	TGFB2	Malaria	APTX_KO_NS vs. APTX_KO_IS	1.79600	0.04724
ENST00000413366	PRKCA	African trypanosomiasis	APTX_KO_NS vs. APTX_KO_IS	1.40287	0.03644

### 2.2 Cytosolic DNA-sensing is dysregulated in APTX KO cells

**Figure 3:**
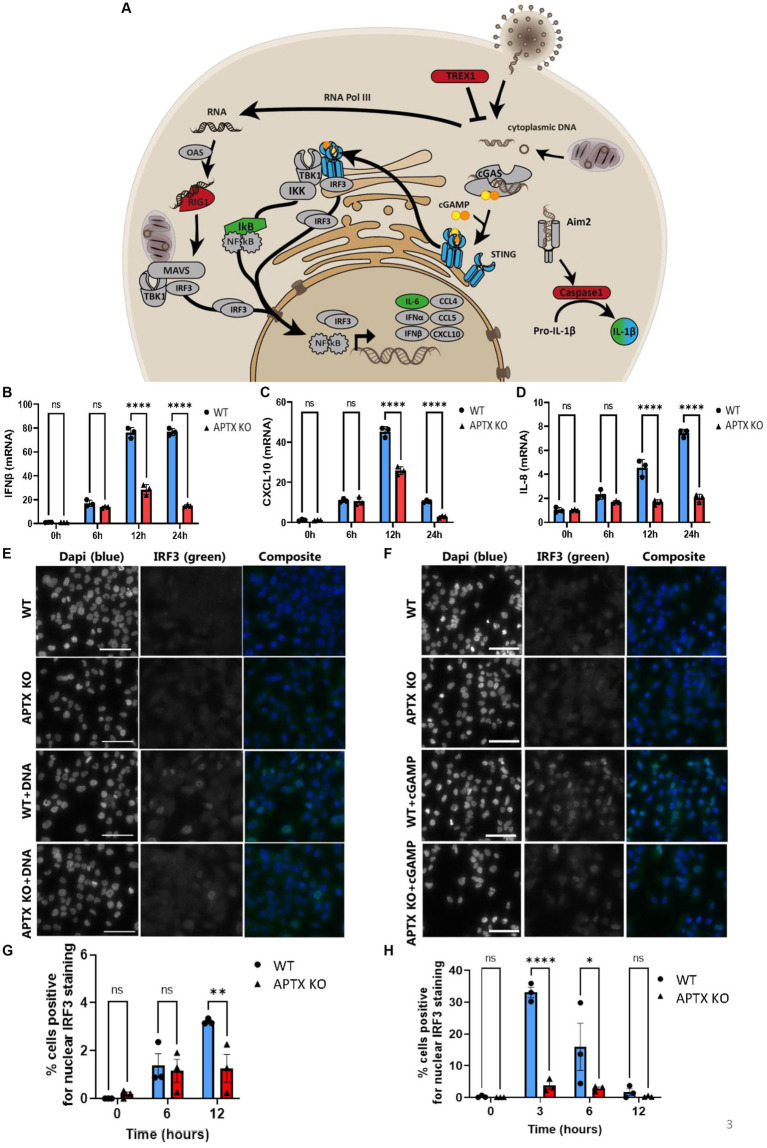
APTX KO affects cytosolic DNA immune stimulation. **(A)** Cytosolic DNA sensing pathways affected by APTX KO and IS. Rendered KEGG pathway plots in Supplementary Figure S1 identified genes that were down- (Red) and up- (green) regulated in APTX KO cells compared to WT (*q* < 0.05). With immune stimulation many similar genes are upregulated in both cell lines, but those that were uniquely upregulated in WT but not in APTX KO cells are marked blue (*q* < 0.05). Post DNA stimulation, RNA was harvested from cells and the levels of IFNβ **(B)**, CXCL10 **(C)** and IL-8 **(D)** were quantified. Cells were also stained for IRF (green) post cytosolic DNA stimulation, and microscope images **(E)** quantified for nuclear IRF translocation **(G)**. Similarly, cells were stimulated with cGAMP, and microscope images **(F)** were quantified for nuclear IRF translocation **(H)**. Scalebar = 50 μm. Graphs show mean ± SEM, analyzed by two-way ANOVA, where significance is scored as ns *p* > 0.05, *p < 0.05, **p < 0.01, ***p < 0.001.

Enrichment analysis identified a dysregulated response to viral infections and a dysregulated innate immune response in APTX deficient cells (Figures 2B,C). This suggests altered cytosolic DNA-sensing dynamics in the absence of functional APTX. Therefore, genes present in DNA-sensing pathways such as cGAS-STING signaling were further investigated. Genes, which were significantly (*q <* 0.05) DE under different APTX and IS statuses were overlaid with the KEGG cytosolic DNA-sensing pathway (Figure 3A; Supplementary Figure S1). The KEGG pathway plot for DE genes with NS in WT vs. KO identified a significant decrease in the expression of TREX1, CASP1, IL-18 and RIG-I when APTX was knocked out (Supplementary Figure S1A). Both TREX1 and RIG-I directly recognize cytosolic DNA and RNA, respectively, therefore lower expression of these genes suggests that DNA- and RNA-sensing is downregulated and dysregulated upon APTX KO. In WT cells the KEGG pathway plot identified lower expression of many cytosolic DNA-sensing genes when there was no stimulation compared to immune stimulated WT cells (Supplementary Figure S1B), which is expected as stimulation with DNA should trigger DNA-sensing pathways and result in higher expression of these genes. Interestingly, when stimulated with dsDNA, RIG-I expression was significantly increased in WT cells despite RNA being the specific substrate for RIG-I. The results were similar in KO cell lines, however, there are a few notable differences. Expression of IL-1B and STING are not significantly affected by IS in APTX KO cells, but ADAR1 and IkB are increased (Supplementary Figure S1). This data demonstrates that when APTX is knocked out, STING is not significantly activated in the presence of cytosolic DNA, illustrating a dysregulation of DNA- and RNA-sensing pathways in APTX KO cells at the transcript level.

### 2.3 *In vitro* analysis of innate immune defects in APTX KO cells

To complement the RNA-seq analysis and confirm phenotypes observed at the transcript level, immune response genes (IFN-β, CXCL10 and IL-8) transcribed downstream of IRF3, following STING activation, were analyzed by qPCR in WT and APTX KO cells after DNA stimulation (Figures 3B–D).

After 12 h of consecutive IS, the transcription of immune response genes was significantly higher in WT cells and continued to be significantly higher 24 h after stimulation compared to the APTX KO cells (Figures 3B–D), showing a clear defect in the DNA-sensing pathway in the APTX KO cells. This supports our RNA-seq analysis as both CXCL10 and IFN-β transcription are significantly elevated after IS in both WT and KO cells compared to NS WT and KO cells (Supplementary Figures S1B,C). IL-8 is not part of the KEGG cytosolic DNA-sensing pathway and is not significantly up- or downregulated following IS in WT or KO cells (data not shown).

To expand the investigation into possible defective activation of the cGAS-STING pathway, microglia cells were transfected with dsDNA (Figure 3E) before they were fixed and stained for IRF3 and scored for IRF3 translocation using macro-enabled image analysis (Figure 3G). DNA stimulation resulted in significantly more IRF3-positive nuclei in WT compared to APTX KO cells at the 12 h time point. DNA treatment for 12 h resulted in phosphorylated of NF-κB which did not differ significantly between WT and APTX-KO cells (Supplementary Figure S2). To avoid this effect being a result of compromised transfection efficiency rather than cGAS activation by dsDNA, the STING agonist cGAMP, which can be taken up from the microenvironment (Su et al., 2022), was added to the cells (Figure 3F). cGAMP treatment, as expected, resulted in a quicker activation of the cGAS-STING pathway as measured by IRF3 translocation into the nucleus, with a significantly higher amount of IRF3 translocation at the 3 h and 6 h time points in WT cells compared to APTX KO cells (Figure 3H). This demonstrates that APTX KO cells have a reduced ability to activate cGAS-STING signaling, which likely leads to a compromised innate immune response.

**Figure 4:**
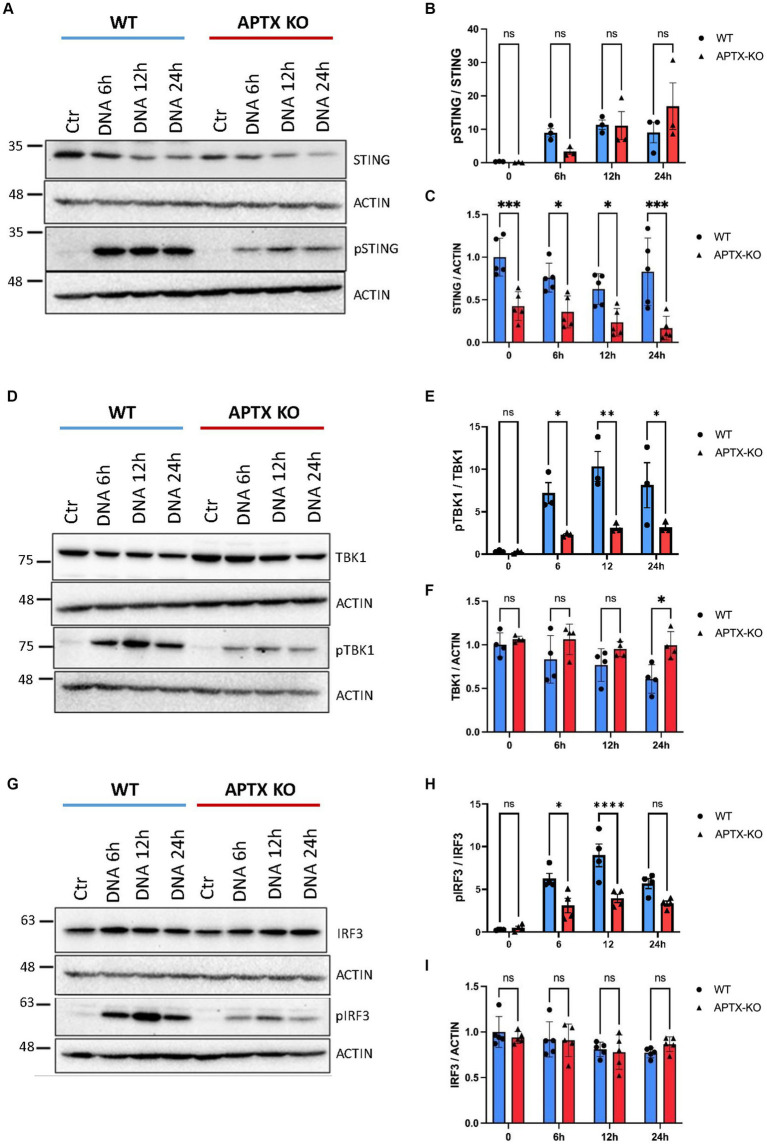
Protein levels of key cGAS-STING effector proteins are affected by APTX KO during dsDNA stimulation. Microglia cells were transfected with dsDNA and harvested for western blot analysis. Blots were stained for STING and pSTING **(A)** and levels quantified in **(B,C)**, TBK1 and pTBK1 **(D)** and levels quantified in **(E,F)** and for IRF3 and pIRF3 **(G)** quantified in **(H,I)**. Graphs show mean ± SEM, analyzed by two-way ANOVA, where significance is scored as ns *p* > 0.05, *p < 0.05, **p < 0.01, ***p < 0.001.

Cell lysates were also harvested after DNA stimulation to investigate levels before and after activation of key cGAS-STING proteins STING, TBK1 and IRF3 (Figure 4). Immunoblots show lower basal and phosphorylated STING (pSTING) in KO cells (Figures 4A,C), with pSTING peaking at 12 h post dsDNA transfection in both WT and KO cells. Although there were lower STING (Figure 4C) and pSTING (Figure 4A) levels in KO cells, the pSTING/STING ratio is similar between APTX and WT cells (Figure 4B). In contrast, although basal levels of TBK1 and IRF3 are similar in WT and KO cells (Figures 4D,F–H), there were significantly higher activation of TBK1 and IRF3 by phosphorylation in WT compared to KO cells at 6 h and 12 h after stimulation, and significantly higher pTBK1 level 24 h after stimulation (Figures 4D,E,G,H). This biochemical data supports the RNA-seq data, showing that APTX KO cells are defective in cGAS-STING signalling across multiple macromolecules, and points to the dysregulation of DNA-sensing potentially stemming from expression of STING.

**Figure 5:**
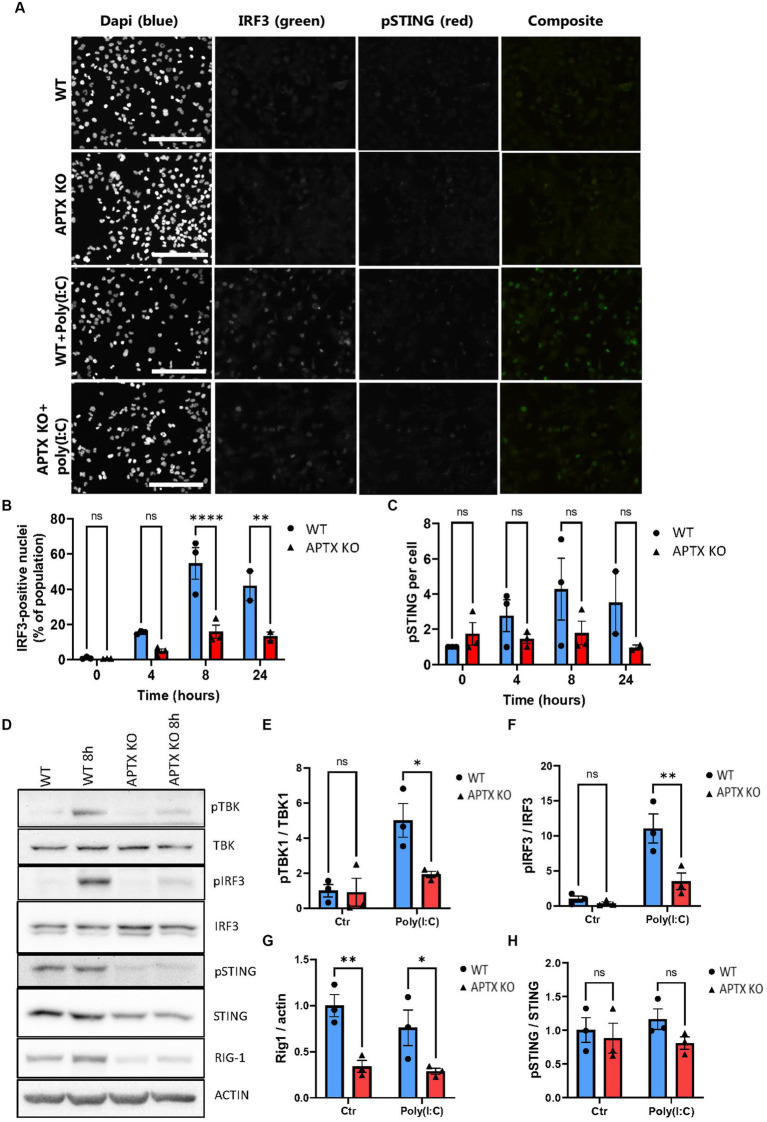
APTK KO affects RNA sensing and immune stimulation. **(A)** Microglia cells were stimulated with the RNA mimic poly(I:C) and stained for IRF3 (green) and pSTING (red), quantified in **(B,C)** respectively. Western blot of key proteins in innate immune sensing pathways TBK, IRF3, STING, and RIG-I were blotted in **(D)** and quantified in **(E–H)**. Scalebar = 250 μm. Graphs show mean ± SEM, analyzed by two-way ANOVA, where significance is scored as ns *p* > 0.05, *p < 0.05, **p < 0.01, ***p < 0.001.

Since several genes related to the RNA-sensing pathway RIG-I/MAVS showed downregulation in APTX KO cells, including DDX58 (RIG-I) (Figures 2D, 3A), microglia cells were treated with the dsRNA-mimic Polyinosinic-polycytidylic acid [poly(I:C)] (Figure 5), which activates RIG-I (Dauletbaev et al., 2015). Similar to dsDNA-stimulation, IRF3 activation was significantly lower in APTX KO cells compared to WT at both 8- and 24 h post poly(I:C) treatment (Figures 5A,B), suggesting defective RNA-sensing when APTX is knocked out. STING was not significantly phosphorylated upon poly(I:C) treatment as expected (Figures 5A,C), as STING is not generally activated downstream of RIG-I (Figure 1B; Yamashiro et al., 2020), even though crosstalk between these pathways is recognized in certain cases (Zevini et al., 2017).

Proteins activated in the RIG-I/MAVS pathway were investigated to further understand the dysregulation of RNA-sensing in APTX KO conditions. Basal TBK1 and IRF3 expression were similar between WT and KO cell lines, however there was significantly higher activation by phosphorylation in WT microglia (Figures 5D–F), the same as observed in response to dsDNA stimulation (Figure 4). Furthermore, the basal level of RIG-I was significantly higher in WT cells compared to APTX KO cells (Figures 5D,G), which is in line with the RNA-seq data showing a downregulation of RIG-I transcript in APTX KO cells compared to WT (Figure 3A; Supplementary Figure S1A). As a control, the phosphorylation of STING was not changed between poly(I:C) treated and control cells, as STING activation is specific for DNA stimulation (Figures 5D,H).

These results support a general innate immune deficiency in the APTX KO microglia cells as expression of critical effectors (STING and RIG-I) are significantly downregulated both before and after stimulation.

### 2.4 AOA patient transcriptomes have altered viral and immune responses

To provide support that the results we present here are clinically relevant, public databases were searched for related publicly available RNA-seq data. This search identified study E-GEOD-124412 (Zheng et al., 2019), which we analyzed below.

**Figure 6:**
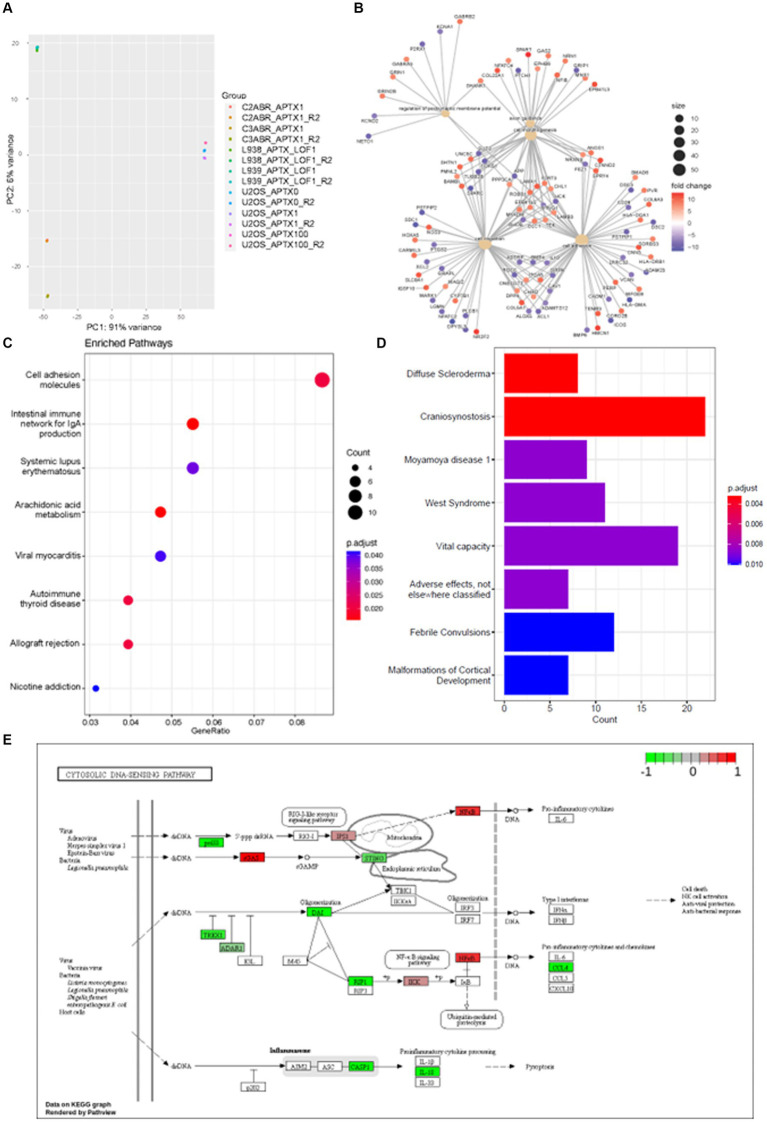
APTX LOF in patients affects immune response and cytosolic DNA- and RNA-sensing. **(A)** PCA plot of sample clustering in study E-GEOD-124412. Functional enrichment plots for networks enrichment genes identified as significantly (*q* < 0.05) differentially expressed genes belonging to network modules in APTX proficient vs. APTX patient LOF cells comparisons where **(B)** shows conserved non-coding elements, **(C)** shows a dot-plot of enriched pathways, and **(D)** shows a barplot of gene sets enriched pathways. **(E)** KEGG plot of genes which were Log2FC > 4 and significantly (*q* < 0.0005) differentially expressed in APTX proficient cells vs. APTX patient LOF cells.

E-GEOD-124412 looked at the impact of APTX on the transcriptome using various conditions and cell lines: U2OS APTX KO; APTX overexpressing U2OS; APTX LOF patient derived lymphoblast cell lines (L938, L939); APTX proficient cell lines (C2ABR and C3ABR). We first observed that samples clustered according to cell line, and patient APTX LOF and U2OS APTX KO cells did not cluster together (Figure 6A).

DE genes were filtered by significance (*q* < 0.0005) and Log2FC (> 4) before further analysis was performed. Comparisons of the APTX proficient healthy cell lines and LOF cell lines identified that enriched pathways in the CNEs affected by APTX LOF are axon guidance, cell morphology, regulation of postsynaptic membrane potential, cell migration, and altered cell adhesion (Figure 6B), with many DE genes upregulated and downregulated in patient cells. Some DE genes are involved in immune signaling processes (HLAs), whilst others are involved in extracellular matrix formation and maintenance (COLs, ADAMs, DSCs). Those involved in synaptic membrane potential are generally involved in ion homeostasis, and those involved in cell migration are associated with cytoskeletal proteins and processes. These changes are also reflected in the enriched KEGG pathways, many enriched in proficient cells relating to the immune system [immune network, lupus, autoimmune thyroid disease, allograft rejection (Figure 6C)], demonstrating an altered immune environment in APTX LOF cells. Enriched gene sets identify a range of what could be classified as neurological disorders [convulsions, vital capacity, cortical development, craniosynostosis (Figure 6D)]. Although there are some clear differences identified in transcriptome changes in LOF patient cells when comparing them to our KO transcriptomes, there are also similarities as pathways relating to the immune system are enriched in WT control cells when APTX is knocked out (Figure 2B) or APTX function is lost (Figure 6C).

Plotting of significantly DE genes (*q* < 0.0005, Log2FC > 4) involved in the KEGG cytosolic DNA-sensing pathway identified that most significantly changed genes involved in DNA-sensing pathways are upregulated in APTX LOF cells compared to healthy cells, including STING and TREX1 (Figure 6E). Interestingly, cGAS expression is downregulated in LOF cells compared to healthy control cells. Furthermore, RIG-I is not significantly impacted by APTX status (Figure 6E) like we see in our KO transcriptome (Figure 3A) or protein data (Figure 5D), but IPS-1 (MAVS) and NF-kB are downregulated in LOF cells. Downregulation of NF-kB in LOF cells could be due to other factors unrelated to RIG-I as NF-kB is involved in many cellular processes (Liu et al., 2017). While these results do not recapitulate exactly what we see in our APTX KO KEGG pathway analysis (Figure 3A; Supplementary Figure S1), it does demonstrate dysregulated cytosolic DNA-sensing when APTX function is lost. Additionally, altered immune responses are observed in the LOF cells (Figures 6B–D) similar to that seen in the KO cells (Figures 2B,C).

Together this data presents the multifaceted roles of APTX and highlights its involvement in cytosolic DNA- and RNA-sensing and innate immunity at both the transcriptional and translational levels.

## 3 Discussion

Dysregulation of the cGAS-STING pathway can lead to autoimmune and inflammatory diseases (Cai et al., 2014; Sun and Hornung, 2022), a process, which can be mediated by the presence of self- and foreign DNA in the cytosol (Gregg et al., 2019; Akbari et al., 2021). Activation of the cGAS-STING pathway plays a central role in immune response pathways, regulating autophagy, and promoting senescence in damaged cells, all of which are important mechanisms for dealing with damaged cells and cancers (Akbari et al., 2021). This study aimed to investigate the innate immune response in AOA1 patients. AOA1 and AT are the most similar ataxias in terms of molecular features and symptoms including association with immunodeficiency (Bras et al., 2015; Paucar et al., 2019; Kato et al., 2021). It is therefore likely that APTX activity influences innate immune response, for instance, following the presence of cytosolic DNA and viral RNA.

RNA and mRNA splicing are affected by APTX KO, with the most enriched process being RNA splicing via transesterification, using bulged adenosine as a nucleophile (Figure 2C). Bulged adenosine in an intron acts as the nucleophile during the first step of splicing (CHU et al., 1998) leading to a global increase in splicing events, which could explain the large number of transcripts DE in APTX KO cell lines compared to WT observed in Supplementary Table S1. Of the DE enriched genes, RBM3 and HNRNPH1 were the only two with lower expression in WT compared to APTX KO microglia (Figure 2C). Expression of RBM3 occurs in response to cold shock and low oxygen tension and is known to reduce the abundance of microRNAs and enhance the phosphorylation of translation initiation factors (Smart et al., 2007). Similarly, HNRNPH1 participates in RNA editing, modification and stability, is a regulator of cellular proliferation, and is frequently upregulated in cancers (Liu et al., 2021). Thus, upregulation of these two genes in APTX KO cells is suggestive of cellular stress and altered gene regulation at the RNA level, providing further evidence of potential genome-wide transcriptome changes. Given the clinical consequences of LOF mutations in APTX, it is noteworthy that genetic variants in the HNRNP family of genes have been linked to neurodegenerative diseases (Gillentine et al., 2021).

A large number of pathways relating to viral infection, as well as inflammatory and immune pathways, were enriched in WT cells but not in APTX KO cells (Figure 2B). This suggests that APTX functions within the immune response and that loss of APTX leads to a reduced ability to respond to infections, ultimately leading to a less effective immune response. Immune responses to viral infection are mediated by various cytosolic DNA- and RNA-sensing pathways, including cGAS-STING and RIG-I/MAVS. Therefore, we investigated the direction of change in expression of significantly (*q <* 0.05) DE genes in the KEGG cytosolic DNA sensing pathway (Figure 3A). This also contains RIG-I/MAVS as dsDNA can be reverse transcribed to RNA to stimulate this pathway in certain cases (Zevini et al., 2017). Interestingly, STING, one of the key signaling proteins in the cGAS-STING pathway, failed to become upregulated in APTX-KO cells upon stimulation, and the RIG1 baseline level was significantly downregulated in APTX deficient cells (Figure 3A).

To confirm the dysregulation of cGAS-STING and RIG-I/MAVS signaling identified at the transcript level, immunoblotting and immunofluorescence microscopy was performed on microglial cells following IS with various compounds. As IRF3 is downstream of both signaling pathways, we scored its activation by translocation after stimulation with dsDNA, a STING agonist and an RNA mimic. Stimulation of WT and KO cells with dsDNA demonstrated a reduced cGAS-STING response in APTX KO cells, measured by activation of IRF3 (Figures 3E,G). The innate immune system is activated via several pathways to orchestrate a proper response to various stimuli (Figure 1B). As there are several cytosolic receptors, (e.g., TLRs, cGAS, AIM2 Figure 1B), dsDNA may stimulate many of these, including the cGAS-STING pathways. Therefore, we assessed the impact of cGAS-STING activation following stimulation with cGAMP, which is a specific, small molecule STING agonist. In correlation, IFR3 activation is around 10-fold stronger in response to cGAMP compared to DNA, and the stimulation leads to a faster response within 3 h for cGAMP, compared to 12 h for dsDNA (Figures 3E–H). This could be due to dsDNA transfection efficiency or the specificity of cGAMP as some dsDNA is likely to have been detected by other pathways and molecules such as TREX1. These results, coupled with the RNA-seq data, clearly demonstrate a dysregulated and ineffective activation of the cGAS-STING pathway in APTX deficient cells.

To elucidate the cause of reduced cGAS-STING signaling when APTX was knocked out, we performed immunoblotting on STING, TBK1 and IRF3, all of which are integral to successful cGAS-STING signaling. Despite no significant change observed in these three proteins at the transcript level (Figure 3A), we found that the basal level of STING was significantly lower in APTX-KO cells at all timepoints (Figure 4C). This led to significantly lower phosphorylation of proteins downstream of STING at one or more timepoints post dsDNA stimulation (Figures 4E,H). It is clear that the ineffective activation of cGAS-STING signaling is due to aberrations in the early stages of signaling.

Differences in cytosolic RNA-sensing was also observed at the transcript level, specifically RIG-I was downregulated in KO cells (Figure 3A). To investigate if this translated to the protein level, we used the dsRNA analog, poly(I:C), which is often used to model viral infections (Dauletbaev et al., 2015; McGarry et al., 2021). Poly(I:C) can signal via TLRs in the endosome upon uptake, or via cytosolic RNA receptors, such as RIG-I or MDA5 (Dauletbaev et al., 2015). Via transfection we ensured cytoplasmic cell entry in order to study signaling via RIG-I. Significant downregulation of RIG-I expression was confirmed by immunoblotting (Figures 5D,G), which explains the lower activation of the RIG-I/MAVS pathway upon Poly(I:C) stimulation (Figure 5).

Related to the reported mitochondrial defects in APTX deficient cells (Akbari et al., 2015; Zheng et al., 2019), it is interesting to note that RIG-I signaling goes through the mitochondrial antiviral-signaling protein (MAVS), which is anchored into mitochondria and peroxisomes (Castanier et al., 2010; Dixit et al., 2010). Fragmentation of the mitochondrial membrane has been shown to inhibit RIG-I/MAVS signaling, and could, in part, explain the decreased signaling observed in the APTX KO cell line (Castanier et al., 2010). It has also been shown that signaling via RIG-I/MAVS promotes mitochondrial network elongation (Castanier et al., 2010), suggesting that mitochondrial dynamics and immune signaling regulate each other. Thus, decreased RIG-I expression, leading to decreased RIG-I/MAVS signaling, could play a part in the observed mitochondrial defects in APTX-deficient cells. This supports a role for APTX in correct mitochondrial and immune function.

Although the relationship between mitochondrial and immune signaling is well established, it is complicated by multiple signaling pathways. It has been shown that moderate stress to mitochondrial DNA elicited via deficiency of transcription factor A mitochondrial (TFAM) or BAX results in the release of mitochondrial DNA into the cytosol, which engages cGAS and promotes STING-IRF3 interferon signaling (West et al., 2015; Victorelli et al., 2023). Deficiency related to the inner mitochondrial membrane protein optic atrophy type 1 (OPA1) has been shown to trigger an NF-κB-related immune response via TLR9, independently of cGAS (Rodríguez-Nuevo et al., 2018). Even though APTX deficiency does impair OPA1 expression (Zheng et al., 2019), we did not find any significantly different activation of NF-κB between WT and APTX-KO cells in our system (Supplementary Figure S2). It has previously been suggested that mitochondrial fragmentation determines whether defective mitochondria are guided into autophagosomes, to potentially trigger TLR9 after fusion with late endosomes, or that mitochondrial DNA instead leaks into the cytoplasm because these are not cleared by the autophagolysosomal system (Rodríguez-Nuevo et al., 2018). In our previous study we showed that APTX-KO leads to mitochondrial fragmentation, without induction of mitophagy, and that APTX deficiency further hampers mitophagy stimulation via the natural mitophagy inducer Urolithin A (Zheng et al., 2019). However, instead of an induction of cGAS, we find that the cGAS-STING pathway is compromised in APTX-KO cells. Whether or not mitochondria play a direct role in the overall inhibition of the DNA- and RNA-sensing pathways in APTX-compromised cells is interesting and warrants future investigation.

It is important to determine if APTX KO cells behave in the same way as patient LOF cells in order to understand the clinical relevance of our data. Publicly available data was identified for a previous RNA-seq study E-GEOD-124412 (Zheng et al., 2019) which contained a healthy APTX WT cell line and an APTX LOF AOA1 patient cell line. In this study, samples cluster according to cell line (Figure 6A) and not APTX status. This could be due to the differences between cell lines, or the difference of a total KO compared to a LOF.

Similar to our APTX KO analysis, many immune related pathways were identified as enriched in WT APTX proficient cells compared to APTX LOF patient cells (Figure 6C). Although the pathways identified are different between the KO and LOF cells, they commonly show enrichment of immune related pathways in control cells. Furthermore, comparison of the WT APTX proficient cell line and the APTX LOF cell line identify that LOF results in significant change in expression of genes involved in altered axon guidance, cell morphology, postsynaptic membrane potential, cell migration, and cell adhesion (Figure 6B). Despite that no immune system specific processes were identified as an enriched biological process, many genes involved in the immune system are identified. For example, HLAs (HLA-DQA1, HLA-DRB1, HLA-DMA) and cluster of differentiations [CDs – CD28, ICOS (CD278)]. HLA-DQA1 and HLA-DRB1 are significantly DE upregulated in WT healthy cells compared to APTX LOF cells. These two genes code for major histocompatibility complexes (MHC) class I and II proteins involved in antigen presentation in adaptive immune response (Crux and Elahi, 2017). As these genes are downregulated in the APTX LOF cells, this suggests that the immune system of AOA1 patients may not be able to identify infections as well as APTX normal individuals, providing evidence that the immune system of AOA1 patients is likely compromised. Changes in the immune system were further reflected in the enriched pathways analysis (Figures 6C,D). While these are not the same enriched pathways as in the WT versus KO data (Figure 2B), it is clear that the immune system response becomes compromised when APTX function is compromised. In correlation, patients with APTX mutations have reported immunological abnormalities (Kato et al., 2021).

Clinically, patients with APTX deficiency suffer from ataxia with childhood-onset and will usually need a wheelchair by their twenties (Kato et al., 2021). Abnormalities vary among patients, but include disorders characterized by low immunoglobulin levels (hypogammaglobulinemia) and low lymphocytes in the blood (lymphopenia), decreased levels of immune cells such as CD8+ T cells and B cells, together with decreased T cell receptor maturation. Even though these are immune system abnormalities, patients are not characterized as having immunodeficiency, which is a clear phenotype of another DNA repair disease, ataxia telangiectasia (van Os et al., 2017). Interestingly, APTX mutations also lead to mild radiosensitivity, a phenotype likewise shared by ataxia telangiectasia patients (Kato et al., 2021), which could be directly correlated with DNA repair defects.

Even though there is not yet any direct patient-derived evidence of defective DNA- or RNA-sensing related to APTX defects, the decrease in immune cells and immunoglobulins levels could reflect a generally compromised immune system, which correlates with our cellular findings reported here.

In conclusion, analysis of both APTX KO and LOF datasets support defective innate immune responses linked to APTX deficiency, possibly due to dysregulated cytosolic DNA- and RNA- sensing. On top of this, there are more significantly DE genes in the healthy versus LOF data compared to the WT versus KO data despite a more stringent significance cut off (*q* < 0.0005 in LOF analysis and *q* < 0.05 in KO analysis), suggesting that the complexity of APTX function is better suited to LOF or knockdown analysis than KO analysis. Future studies on regulatory pathways upstream the DNA- and RNA- sensing pathways described here would provide valuable insights to further our understanding of the relation between APTX and innate immune sensing.

## 4 Materials and methods

### 4.1 Sample attributes and comparisons

The experimental setup and design are outlined in Table 4.

**Table 4:** Summary information for the samples available, treatments and number of replicates.

Sample ID	Sequencing run	Treatment	Label	Label in text
1–1	1	Control	APTX1_NS	WT_NS
1–2	2	Control	APTX1_NS	WT_NS
1–3	3	Control	APTX1_NS	WT_NS
2–1	1	Immune stimulated	APTX1_IS	WT_IS
2–2	2	Immune stimulated	APTX1_IS	WT_IS
2–3	3	Immune stimulated	APTX1_IS	WT_IS
3–1	1	APTX deletion	APTX0_NS	APTX_KO_NS
3–2	2	APTX deletion	APTX0_NS	APTX_KO_NS
3–3	3	APTX deletion	APTX0_NS	APTX_KO_NS
4–1	1	APTX deletion immune stimulated	APTX0_IS	APTX_KO_IS
4–2	2	APTX deletion immune stimulated	APTX0_IS	APTX_KO_IS
4–3	3	APTX deletion immune stimulated	APTX0_IS	APTX_KO_IS

### 4.2 Generating APTX KO microglial cell lines

HMC3 cells (ATCC CRL-3304) were seeded on 6-well dish in EMEM medium. Next day they were transfected with aprataxin double-nickase plasmid (sc-417083-NIC, Santa Cruz Biotechnology) using Lipofectamine LTX (Invitrogen). 24 h later the cells were cultured in 2 μg/mL puromycin for five days and then in 4 μ/mL puromycin for two days to select for GFP-Cas9 expressing cells. Cells were harvested and reseeded on 150 mm dishes at 20 cells per dish. Single cell colonies were propagated in EMEM without puromycin, followed by WB analysis for APTX expression (Figure 2A).

### 4.3 Cell treatments

pAcGFP1-Hyg-N1 vector (Clontech) was digested using EcoRI and AseI. The digested plasmids were mixed and purified using NucleoSpin Plasmid Mini Kit (Macherey Nagel), resulting in a mixture of DNA fragments of 236, 1,143, 1,604, 2,209, 2,811, and 3,639 bp that were used to stimulate cGAS-STING by transfection, “DNA.”

Cells (1.5 × 10^6^) were seeded on 10-cm dishes. Next day, the cells were transfected with 500 ng digested DNA using Lipofectamine 3,000 (Thermo Fisher Scientific).

For cGAMP-mediated STING stimulation, 30 μM 2′3’-cGAMP (InvivoGen) was added to full medium for the indicated time points. For poly(I:C)-mediated RIG-I stimulation, 0.1 μg/mL (InvivoGen, tlrl-picw) was added to cells using lipofectamine 3,000 (Thermo Fisher Scientific).

### 4.4 RNA isolation and quantitative PCR (qPCR) analysis of gene expression

Total RNA was purified using RNeasy Mini Kit (Qiagen). Complementary DNA was prepared using Maxima Reverse Transcriptase and Oligo dT (12–18) (Thermo Fisher Scientific- Life tech). qPCR was performed using a real-time PCR kit (Bio-Rad1725271) following the manufacturer’s protocol. GraphPad Prism V9.2 was used for plotting and performing two-way ANOVA analysis. Significance is scored as ns *p* > 0.05, * *p* < 0.05, ** *p* < 0.01, *** *p* < 0.001. The number of biological replicates, n, corresponds to the number of datapoints in each figure. The nucleotide sequence of the primers is indicated Table 5.

**Table 5:** Nucleotide sequence of primers.

Primer name	Primer nucleotide sequence
*IFN-β-F*	AAGCAGCAATTTTCAGTGTCAGA
*IFN-β-R*	CCTCAGGGATGTCAAAGTTCA
*CXCL10-F*	TGGCATTCAAGGAGTACCTC
*CXCL10-R*	TTGTAGCAATGATCTCAACACG
*IL-8-F*	TGCCAAGGAGTGCTAAAG
*IL-8-R*	CTCCACAACCCTCTGCAC
*β-actin-F*	GGATGACAGAAGGAGATCACTG
*β-actin-R*	CGATCCACACGGAGTACTTG

### 4.5 Immunofluorescence

HMC3 cells were grown on Poly-L-lysine (P1399, Merck) coated eight-chambered object glass (177,402, Thermo Scientific). The following day cells were treated [DNA/cGAMP/Poly(I:C)] as described above, washed in PBS and fixed for 10 min in 4% paraformaldehyde (sc-281692, Santa Cruz) at room temperature. Cells were blocked in 2% BSA (0332, VWR) dissolved in PBS then permeabilized using 0.1% Triton X-100 (T8787, sigma) in PBS. Primary antibodies: IRF3 (sc-33641, Santa Cruz) and pSTING (50907S, Cell Signaling) were diluted in PBS containing 1% BSA and incubated with cells over night at 4°C followed by washing with PBS. Secondary Alexa488- and Alexa568-conjugated goat-anti-mouse (A11029, Invitrogen) and goat-anti-rabbit (A11011, Invitrogen) antibodies and DAPI (D9542, Merck) were incubated with cells for 1½ h at room temperature. The chamber was removed, and cells were mounted (S3023, Dako). Slides were imaged with an upright Leica DM4B microscope with a Leica EL6000 external light source and a Leica DFC365 camera. Images were analyzed using an ImageJ (Version v1.53m) macro. Briefly, the macro uses the DAPI channel to select the nuclear regions and measures the intensity of the IRF3 signals. The percentage of nuclei positive for IRF3 staining above a certain threshold was calculated using Microsoft Excel software. The pSTING, signal was measured and normalized to the number of cells. GraphPad Prism V9.2 was used for plotting and performing two-way ANOVA analysis. Significance is scored as ns *p* > 0.05, * *p* < 0.05, ** *p* < 0.01, *** *p* < 0.001. The number of biological replicates, n, corresponds to the number of datapoints in each figure.

### 4.6 Western blot

Whole cell extracts were prepared by suspending cell pellets in 20 mM HEPES-KOH, pH 7.5, 150 mM KCl, 10% glycerol, 1% Triton X-100, 1% IGEPAL, 1 mM EDTA, 1 mM DTT, EDTA-free Complete protease inhibitor cocktail (Merck), and PhosSTOP (Merck) and left on ice for 60 min. Cell debris was pelleted at 15,000 g for 15 min, before supernatants were collected.

The extracts were separated in Tris-glycine SDS gels and transferred onto PVDF membrane. Primary antibodies used were STING (13647S), phospho-STING (Ser366, 19781S), TBK1 (3504S), phospho-TBK1 (Ser172, 5483S), and phospho-IRF-3 (Ser386, 37829S) all from Cell Signaling. APTX (sc-374108), IRF3 (SL-12, sc-33641) and Rig1 (D12, sc-376845) antibodies were from Santa Cruz and Actin (A5441) was from Merck. GraphPad Prism V9.2 was used for plotting and performing two-way ANOVA analysis. Significance is scored as ns *p* > 0.05, *p < 0.05, **p < 0.01, ***p < 0.001. The number of biological replicates, *n*, corresponds to the number of datapoints in each figure.

### 4.7 RNA-seq data processing

Available samples, their attributes and an overview of the experimental design are shown in Table 4. Paired end sequencing files for each sample and sequencing run were aligned to the HG38 genome using HISAT2. Salmon (Patro et al., 2017) and DESeq2 (Love et al., 2014) were used to identify DE genes. Ballgown (Fu et al., 2023) was further used to identify DE transcripts and genes. Condition comparisons were made as follows using both the Salmon and the Ballgown workflows: APTX1_NS versus APTX1_IS; APTX1_NS versus APTX0_NS; APTX1_NS versus APTX0_IS; APTX1_IS versus APTX0_IS. Significantly (*q* < 0.05) DE genes and transcripts for each of the comparisons were then identified (Table 2).

### 4.8 RNA-seq data analysis

Significantly (*q* < 0.05) DE genes and transcripts were converted to stable Entrez IDs. Functional enrichment analysis of pathways and conserved non-coding elements was performed on APTX WT and APTX KO microglial cells with NS using R package clusterProfiler (Wu et al., 2021). Hierarchically clustered heatmapping was performed on the expression values of significantly (*q* < 0.05) DE genes and transcripts across all four microglial sample conditions (APTX1_NS, APTX1_IS, APTX0_NS, and APTX0_IS). KEGG pathway analysis was performed on significantly (*q* < 0.05) DE genes within KEGG pathway hsa04623 [cytosolic DNA-sensing pathway – *Homo sapiens* (human)] using R packages KEGGrest (Tenenbaum and Maintainer, 2022) and pathview (Luo and Brouwer, 2013). Data has been submitted to GEO with the accession number (GSE245766).

### 4.9 APTX LOF data identification, RNA-seq processing, and analysis

In Array Express, an additional study was identified with data complementary to this study using key search terms ‘APTX’, ‘Ataxia’ and ‘cGAS-STING’. Study E-GEOD-124412 was identified which investigated APTX loss of function (LOF). Raw paired end fastq files were downloaded and alignment free quantification was achieved using Salmon. DE expressed genes and transcripts were identified in AOA1 patient cell lines (L938 and L939), healthy cell lines (C3ABR and C2ABR) and cancer cell lines (U2OS) using R-package DESeq2.

Factors affecting the expression of genes were assessed using PCA plots which considered the cell line and APTX status. DE expressed genes were filtered for significance (*q* < 0.0005) and Log2FoldChange (> 4) before functional enrichment analysis and KEGG pathway analysis was performed as described above.

### 4.10 Statistical analysis

Error bars represent standard error (SE) as indicated in the figure legends. Data were processed and statistical analyses were performed in GraphPad Prism. Two-way ANOVA analysis were applied. Significance is scored as ns *p* > 0.05, *p < 0.05, **p < 0.01, ***p < 0.001. The number of biological replicates, *n*, is indicated in each figure legend.

## Data availability statement

Original RNA-seq datasets are available in the publicly accessible repository Gene Expression Omnibus (GEO) and can be found at the following link https://www.ncbi.nlm.nih.gov/geo/query/acc.cgi accession number GSE245766. Existing datasets are available in the publicly accessible repository BioStudies (formerly known as Array Express) at accession number E-GEOD-124412.

## Author contributions

HM: Conceptualization, Investigation, Visualization, Writing – original draft, Writing – review & editing. LP: Conceptualization, Investigation, Methodology, Writing – original draft, Writing – review & editing. R-LS: Conceptualization, Investigation, Writing – review & editing. MA: Conceptualization, Investigation, Writing – review & editing. LR: Resources, Supervision, Writing – review & editing. DS: Conceptualization, Methodology, Supervision, Writing – review & editing. VB: Conceptualization, Supervision, Writing – review & editing, Funding acquisition, Investigation, Resources.

## References

[ref1] Ahel I. Rass U. El-Khamisy S. F. Katyal S. Clements P. M. McKinnon P. J.. (2006). The neurodegenerative disease protein aprataxin resolves abortive DNA ligation intermediates. Nature 443, 713–716. doi: 10.1038/nature05164 16964241

[ref2] Airoldi G. Guidarelli A. Cantoni O. Panzeri C. Vantaggiato C. Bonato S.. (2010). Characterization of two novel SETX mutations in AOA2 patients reveals aspects of the pathophysiological role of senataxin. Neuroge. 11, 91–100. doi: 10.1007/S10048-009-0206-0/METRICS, PMID: 19593598

[ref3] Akbari M. Shanley D. P. Bohr V. A. Rasmussen L. J. (2021). Cytosolic self-DNA—A potential source of chronic inflammation in aging. Cells 10:3544. doi: 10.3390/cells10123544, PMID: 34944052 PMC8700131

[ref4] Akbari M. Sykora P. Bohr V. A. (2015). Slow mitochondrial repair of 5′-AMP renders mtDNA susceptible to damage in APTX deficient cells. Sci. Rep. 5:12876. doi: 10.1038/srep12876, PMID: 26256098 PMC4530458

[ref6] Bahat A. MacVicar T. Langer T. (2021). Metabolism and innate immunity meet at the mitochondria. Front. Cell Dev. Biol. 9:720490. doi: 10.3389/FCELL.2021.720490/BIBTEX, PMID: 34386501 PMC8353256

[ref7] Bogeski I. Gulaboski R. Kappl R. Mirceski V. Stefova M. Petreska J.. (2011). Calcium binding and transport by coenzyme Q. J. Am. Chem. Soc. 133, 9293–9303. doi: 10.1021/ja110190t 21548646

[ref8] Bras J. Alonso I. Barbot C. Costa M. M. Darwent L. Orme T.. (2015). Mutations in PNKP cause recessive Ataxia with oculomotor apraxia type 4. Am. J. Hum. Genet. 96, 474–479. doi: 10.1016/j.ajhg.2015.01.005, PMID: 25728773 PMC4375449

[ref9] Cai X. Chiu Y.-H. Chen Z. J. (2014). The cGAS-cGAMP-STING pathway of cytosolic DNA sensing and signaling. Mol. Cell 54, 289–296. doi: 10.1016/j.molcel.2014.03.040, PMID: 24766893

[ref10] Castanier C. Garcin D. Vazquez A. Arnoult D. (2010). Mitochondrial dynamics regulate the RIG-I-like receptor antiviral pathway. EMBO Rep. 11, 133–138. doi: 10.1038/EMBOR.2009.258 20019757 PMC2828750

[ref11] Chu V. T. Liu Q. Podar M. Perlman P. S. And Pyle A. M. (1998). More than one way to splice an RNA: branching without a bulge and splicing without branching in group II introns. RNA 4:S1355838298980724. doi: 10.1017/S1355838298980724, PMID: 9769094 PMC1369692

[ref12] Coutinho P. Barbot C. Coutinho P. (2023). Ataxia with oculomotor apraxia type 1. GeneReviews.® Available at: https://www.ncbi.nlm.nih.gov/books/NBK1456/ (Accessed August 1, 2012).

[ref13] Crux N. B. Elahi S. (2017). Human leukocyte antigen (HLA) and immune regulation: how do classical and non-classical HLA alleles modulate immune response to human immunodeficiency virus and hepatitis C virus infections? Front. Immunol. 8:832. doi: 10.3389/fimmu.2017.00832, PMID: 28769934 PMC5513977

[ref14] Danno S. Nishiyama H. Higashitsuji H. Yokoi H. Xue J. H. Itoh K.. (1997). Increased transcript level of RBM3, a member of the Glycine-rich RNA-binding protein family, in human cells in response to cold stress. Biochem. Biophys. Res. Commun. 236, 804–807. doi: 10.1006/BBRC.1997.7059, PMID: 9245737

[ref15] Dauletbaev N. Cammisano M. Herscovitch K. Lands L. C. (2015). Stimulation of the RIG-I/MAVS pathway by Polyinosinic:Polycytidylic acid upregulates IFN-β in airway epithelial cells with minimal Costimulation of IL-8. J. Immunol. 195, 2829–2841. doi: 10.4049/JIMMUNOL.1400840 26283481

[ref16] Dixit E. Boulant S. Zhang Y. Lee A. S. Y. Odendall C. Shum B.. (2010). Peroxisomes are signaling platforms for antiviral innate immunity. Cells 141, 668–681. doi: 10.1016/J.CELL.2010.04.018, PMID: 20451243 PMC3670185

[ref17] Dresios J. Aschrafi A. Owens G. C. Vanderklish P. W. Edelman G. M. Mauro V. P. (2005). Cold stress-induced protein Rbm3 binds 60S ribosomal subunits, alters microRNA levels, and enhances global protein synthesis. Proc. Natl. Acad. Sci. 102, 1865–1870. doi: 10.1073/pnas.0409764102, PMID: 15684048 PMC548588

[ref18] Ehlén Å. Brennan D. J. Nodin B. O’Connor D. P. Eberhard J. Alvarado-Kristensson M.. (2010). Expression of the RNA-binding protein RBM3 is associated with a favourable prognosis and cisplatin sensitivity in epithelial ovarian cancer. J. Transl. Med. 8:78. doi: 10.1186/1479-5876-8-78 20727170 PMC2936876

[ref19] Fang E. F. Scheibye-Knudsen M. Brace L. E. Kassahun H. Sengupta T. Nilsen H.. (2014). Defective mitophagy in XPA via PARP-1 hyperactivation and NAD(+)/SIRT1 reduction. Cells 157, 882–896. doi: 10.1016/J.CELL.2014.03.026, PMID: 24813611 PMC4625837

[ref20] Fu J. Frazee A. C. Collado-Torres L. Jaffe A. E. Leek J. T. (2023). Ballgown: flexible, isoform-level differential expression analysis. R package version 2.32.0. Available at: https://bioconductor.org/packages/ballgown

[ref21] Fusco G. Chen S. W. Williamson P. T. F. Cascella R. Perni M. Jarvis J. A.. (2017). Structural basis of membrane disruption and cellular toxicity by a-synuclein oligomers. Science 358, 1440–1443. doi: 10.1126/science.aan6160 29242346

[ref22] Gillentine M. A. Wang T. Hoekzema K. Rosenfeld J. Liu P. Earl R. K.. (2021). Rare deleterious mutations of HNRNP genes result in shared neurodevelopmental disorders. Genome Med. 13:63. doi: 10.1186/s13073-021-00870-6, PMID: 33874999 PMC8056596

[ref23] Gregg R. W. Sarkar S. N. Shoemaker J. E. (2019). Mathematical modeling of the cGAS pathway reveals robustness of DNA sensing to TREX1 feedback. J. Theor. Biol. 462, 148–157. doi: 10.1016/j.jtbi.2018.11.001, PMID: 30395807

[ref24] Hou Y. Wei Y. Lautrup S. Yang B. Wang Y. Cordonnier S.. (2021). NAD+ supplementation reduces neuroinflammation and cell senescence in a transgenic mouse model of Alzheimer’s disease via cGAS-STING. Proc. Natl. Acad. Sci. U. S. A. 118:e2011226118. doi: 10.1073/PNAS.2011226118/-/DCSUPPLEMENTAL 34497121 PMC8449423

[ref1002] Jacobi H. Minnerop M. Klockgether T. (2013). The genetics of spinocerebellar ataxias. Der Nervenarzt. 84, 137–142. doi: 10.1007/S00115-012-3637-Z, PMID: 23338152

[ref25] Kato T. Tamura Y. Matsumoto H. Kobayashi O. Ishiguro H. Ogawa M.. (2021). Immunological abnormalities in patients with early-onset ataxia with ocular motor apraxia and hypoalbuminemia. Clin. Immunol. 229:108776. doi: 10.1016/j.clim.2021.108776, PMID: 34118401

[ref26] Kijas A. W. Harris J. L. J. M. Harris J. L. J. M. Lavin M. F. (2006). Aprataxin forms a discrete branch in the HIT (histidine triad) superfamily of proteins with both DNA/RNA binding and nucleotide hydrolase activities. J. Biol. Chem. 281, 13939–13948. doi: 10.1074/jbc.M507946200 16547001

[ref27] Klockgether T. (2007). Ataxias. Parkinsonism Relat. Disord. 13, S391–S394. doi: 10.1016/S1353-8020(08)70036-1 18267270

[ref28] Liu M. Yang L. Liu X. Nie Z. Zhang X. Lu Y.. (2021). HNRNPH1 is a novel regulator of cellular proliferation and disease progression in chronic myeloid leukemia. Front. Oncol. 11:2859. doi: 10.3389/fonc.2021.682859, PMID: 34295818 PMC8290130

[ref29] Liu T. Zhang L. Joo D. Sun S. C. (2017). NF-κB signaling in inflammation. Signal Transduct. Target. Ther. 2:17023. doi: 10.1038/sigtrans.2017.23, PMID: 29158945 PMC5661633

[ref30] Love M. I. Huber W. Anders S. (2014). Moderated estimation of fold change and dispersion for RNA-seq data with DESeq2. Genome Biol. 15, 1–21. doi: 10.1186/S13059-014-0550-8/FIGURES/9 PMC4302049 25516281

[ref31] Luo W. Brouwer C. (2013). Pathview: an R/Bioconductor package for pathway-based data integration and visualization. Bioinformatics 29, 1830–1831. doi: 10.1093/BIOINFORMATICS/BTT285, PMID: 23740750 PMC3702256

[ref32] Matsuda A. Ogawa M. Yanai H. Naka D. Goto A. Ao T.. (2011). Generation of mice deficient in RNA-binding motif protein 3 (RBM3) and characterization of its role in innate immune responses and cell growth. Biochem. Biophys. Res. Commun. 411, 7–13. doi: 10.1016/j.bbrc.2011.06.038, PMID: 21684257

[ref33] McGarry N. Murray C. L. Garvey S. Wilkinson A. Tortorelli L. Ryan L.. (2021). Double stranded RNA drives anti-viral innate immune responses, sickness behavior and cognitive dysfunction dependent on dsRNA length, IFNAR1 expression and age. Brain Behav. Immun. 95, 413–428. doi: 10.1016/J.BBI.2021.04.016 33892139 PMC8447494

[ref34] Moreira M.-C. C. Barbot C. Tachi N. Kozuka N. Uchida E. Gibson T.. (2001). The gene mutated in ataxia-ocular apraxia 1 encodes the new HIT/Zn-finger protein aprataxin. Nat. Genet. 29, 189–193. doi: 10.1038/ng1001-189 11586300

[ref35] Neckles C. Boer R. E. Aboreden N. Cross A. M. Walker R. L. Kim B.-H.. (2019). HNRNPH1-dependent splicing of a fusion oncogene reveals a targetable RNA G-quadruplex interaction. RNA 25, 1731–1750. doi: 10.1261/rna.072454.119, PMID: 31511320 PMC6859848

[ref36] Papadimitriou A. Hadjigeorgiou G. M. Divari R. Papagalanis N. Comi G. Bresolin N. (1996). The influence of coenzyme Q10 on total serum calcium concentration in two patients with kearns-Sayre syndrome and hypoparathyroidism. Neuromuscul. Disord. 6, 49–53. doi: 10.1016/0960-8966(95)00020-8 8845718

[ref37] Pasmanter N. Iheanacho F. Hashmi M. F. (2023). Biochemistry, Cyclic GMP. 31194391

[ref38] Patro R. Duggal G. Love M. I. Irizarry R. A. Kingsford C. (2017). Salmon provides fast and bias-aware quantification of transcript expression. Nat. Methods 14, 417–419. doi: 10.1038/nmeth.4197 28263959 PMC5600148

[ref39] Paucar M. Malmgren H. Taylor M. Reynolds J. J. Svenningsson P. Press R.. (2016). Expanding the ataxia with oculomotor apraxia type 4 phenotype. Neurol. Genet. 2:e49. doi: 10.1212/NXG.0000000000000049, PMID: 27066586 PMC4817910

[ref40] Paucar M. Taylor A. M. R. Hadjivassiliou M. Fogel B. L. Svenningsson P. (2019). Progressive Ataxia with elevated alpha-fetoprotein: diagnostic issues and review of the literature. Tremor Other Hyperkinet Mov (N Y) 9:708. doi: 10.7916/tohm.v0.708, PMID: 31656689 PMC6790008

[ref41] Quinzii C. M. Kattah A. G. Naini A. Akman H. O. Mootha V. K. DiMauro S.. (2005). Coenzyme Q deficiency and cerebellar ataxia associated with an aprataxin mutation. Neurology 64, 539–541. doi: 10.1212/01.WNL.0000150588.75281.58, PMID: 15699391

[ref42] Rass U. Ahel I. West S. C. (2007). Actions of Aprataxin in multiple DNA repair pathways. J. Biol. Chem. 282, 9469–9474. doi: 10.1074/jbc.M611489200, PMID: 17276982

[ref43] Rodríguez-Nuevo A. Díaz-Ramos A. Noguera E. Díaz-Sáez F. Duran X. Muñoz J. P.. (2018). Mitochondrial DNA and TLR9 drive muscle inflammation upon Opa1 deficiency. EMBO J. 37:e96553. doi: 10.15252/EMBJ.201796553 29632021 PMC5978453

[ref44] Rudenskaya G. E. Marakhonov A. V. Shchagina O. A. Lozier E. R. Dadali E. L. Akimova I. A.. (2019). Ataxia with oculomotor apraxia type 4 with PNKP common “Portuguese” and novel mutations in two Belarusian families. J Pediatr Genet 8, 058–062. doi: 10.1055/S-0039-1684008, PMID: 31061747 PMC6499616

[ref45] Sano Y. Date H. Igarashi S. Onodera O. Oyake M. Takahashi T.. (2004). Aprataxin, the causative protein for EAOH is a nuclear protein with a potential role as a DNA repair protein. Ann. Neurol. 55, 241–249. doi: 10.1002/ana.10808, PMID: 14755728

[ref46] Smart F. Aschrafi A. Atkins A. Owens G. C. Pilotte J. Cunningham B. A.. (2007). Two isoforms of the cold-inducible mRNA-binding protein RBM3 localize to dendrites and promote translation. J. Neurochem. 101, 1367–1379. doi: 10.1111/J.1471-4159.2007.04521.X 17403028

[ref47] Su M. Zheng J. Gan L. Zhao Y. Fu Y. Chen Q. (2022). Second messenger 2′3′-cyclic GMP–AMP (2′3′-cGAMP): synthesis, transmission, and degradation. Biochem. Pharmacol. 198:114934. doi: 10.1016/J.BCP.2022.114934, PMID: 35104477

[ref48] Sun Z. Hornung V. (2022). cGAS–STING signaling. Curr. Biol. 32, R730–R734. doi: 10.1016/j.cub.2022.05.027 35820380

[ref49] Szklarczyk D. Gable A. L. Nastou K. C. Lyon D. Kirsch R. Pyysalo S.. (2021). The STRING database in 2021: customizable protein–protein networks, and functional characterization of user-uploaded gene/measurement sets. Nucleic Acids Res. 49, D605–D612. doi: 10.1093/nar/gkaa1074, PMID: 33237311 PMC7779004

[ref50] Tada M. Yokoseki A. Sato T. Makifuchi T. Onodera O. (2010). Early-onset Ataxia with ocular motor apraxia and hypoalbuminemia/Ataxia with oculomotor apraxia. Adv. Exp. Med. Biol., 21–33. doi: 10.1007/978-1-4419-6448-9_3 20687492

[ref5] Tassan N. Khalil D. Shinwari J. Al Sharif L. Bavi P. Abduljaleel Z.. (2012). A missense mutation in PIK3R5 gene in a family with ataxia and oculomotor apraxia. Hum. Mutat. 33, 351–354. doi: 10.1002/HUMU.21650, PMID: 22065524

[ref51] Tenenbaum D. Maintainer B. (2022). Client-side REST access to the Kyoto encyclopedia of genes and genomes (KEGG). KEGGREST. doi: 10.18129/B9.bioc.KEGGREST

[ref52] Tumbale P. Williams J. S. Schellenberg M. J. Kunkel T. A. Williams R. S. (2014). Aprataxin resolves adenylated RNA–DNA junctions to maintain genome integrity. Nature 506, 111–115. doi: 10.1038/nature12824 24362567 PMC4064939

[ref53] van Os N. J. H. Jansen A. F. M. van Deuren M. Haraldsson A. van Driel N. T. M. Etzioni A.. (2017). Ataxia-telangiectasia: immunodeficiency and survival. Clin. Immunol. 178, 45–55. doi: 10.1016/J.CLIM.2017.01.009 28126470

[ref54] Victorelli S. Salmonowicz H. Chapman J. Martini H. Vizioli M. G. Riley J. S.. (2023). Apoptotic stress causes mtDNA release during senescence and drives the SASP. Nature 622, 627–636. doi: 10.1038/S41586-023-06621-4, PMID: 37821702 PMC10584674

[ref1003] Walker J. L. Chamberlain S. Robinson N. (1980). Lipids and lipoproteins in Friedreich’s ataxia. J. Neuro. Neurosur. Psych. 43, 111–117. doi: 10.1136/jnnp.43.2.111, PMID: 7359148 PMC490484

[ref55] West A. P. Khoury-Hanold W. Staron M. Tal M. C. Pineda C. M. Lang S. M.. (2015). Mitochondrial DNA stress primes the antiviral innate immune response. Nature 520, 553–557. doi: 10.1038/NATURE14156, PMID: 25642965 PMC4409480

[ref56] Wu T. Hu E. Xu S. Chen M. Guo P. Dai Z.. (2021). clusterProfiler 4.0: a universal enrichment tool for interpreting omics data. The Innovation 2:100141. doi: 10.1016/J.XINN.2021.100141, PMID: 34557778 PMC8454663

[ref57] Yamashiro L. H. Wilson S. C. Morrison H. M. Karalis V. Chung J. Y. J. Chen K. J.. (2020). Interferon-independent STING signaling promotes resistance to HSV-1 *in vivo*. Nat. Commun. 11:17156. doi: 10.1038/s41467-020-17156-x, PMID: 32636381 PMC7341812

[ref58] Zang X. Chen S. Zhu J. Y. Ma J. Zhai Y. (2022). The emerging role of central and peripheral immune systems in neurodegenerative diseases. Front. Aging Neurosci. 14:872134. doi: 10.3389/FNAGI.2022.872134/BIBTEX 35547626 PMC9082639

[ref59] Zevini A. Olagnier D. Hiscott J. (2017). Cross-talk between the cytoplasmic RIG-I and STING sensing pathways. Trends Immunol. 38, 194–205. doi: 10.1016/J.IT.2016.12.004 28073693 PMC5329138

[ref60] Zheng J. Croteau D. L. Bohr V. A. Akbari M. (2019). Diminished OPA1 expression and impaired mitochondrial morphology and homeostasis in Aprataxin-deficient cells. Nucleic Acids Res. 47, 4086–4110. doi: 10.1093/nar/gkz083, PMID: 30986824 PMC6486572

[ref61] Zhong Y. Kinio A. Saleh M. (2013). Functions of NOD-like receptors in human diseases. Front. Immunol. 4:67819. doi: 10.3389/FIMMU.2013.00333 PMC3797414 24137163

[ref62] Zhou R.-B. Lu X.-L. Zhang C.-Y. Yin D.-C. Zhou R.-B. Lu X.-L.. (2017). RNA binding motif protein 3: a potential biomarker in cancer and therapeutic target in neuroprotection. Oncotarget 8, 22235–22250. doi: 10.18632/ONCOTARGET.14755, PMID: 28118608 PMC5400660

