# Supplementary material for: The DNA repair enzyme, aprataxin, plays a role in innate immune signaling
Source: Front Aging Neurosci. 2023 Dec 15;15:1290681. doi: 10.3389/fnagi.2023.1290681 (PMC10754971; doi:10.3389/fnagi.2023.1290681)
Supplement: Supplementary file 1 [file Data_Sheet_1.pdf]

frontiers

Supplementary Material

Article Title

Helena B. Madsen[1+], Louise I. Pease[2+], Rebekah-Louise Scanlan[2], Mansour Akbari[1], Lene J. Ramussen[1], Daryl P. Shanley[2*], Vilhelm A. Bohr[1*]

[1]University of Copenhagen, Department of Cellular and Molecular Medicine, 2200 Copenhagen N, Denmark

[2]CAMPUS for Ageing and Vitality, Newcastle University, Newcastle, NE4 6BE, United Kingdom

[*]corresponding authors: vbohr@sund.ku.dk and daryl.shanley@newcastle.ac.uk

[+]these authors contributed equally to this work.

1 Supplementary Figures and Tables

1.1 **Supplementary Table 1:** Ballgown transcript analysis showing the number of significantly (q < 0.05) differentially expressed transcripts.

Comparisons	Genes	Transcripts
WT NS vs WT IS	0	603
WT NS vs APTX KO NS	700	3611
WT NS vs APTX KO IS	5512	5064
WT IS vs APTX KO IS	814	3780
APTX KO NS vs APTX KO IS	657	402

1.2 **Supplementary Table 2**: Summary of transcripts significantly (q < 0.05) DE in response to immune stimulation which are in the same KEGG pathways, but the transcripts DE are different in WT and KO mutant comparisons.

KEGG Term	Comparison	Transcript	Gene	Comparison	Transcript	Gene
Chagas disease (American trypanosomiasis)	APTX_KO_NS vs APTX_KO_IS	ENST00000395148	CFLAR	WT_NS vs WT_IS	ENST00000248244	TICAM1

Hepatitis C	APTX_KO_NS vs APTX_KO_IS	ENST00000379883	DDX58 (RIG-1)	WT_NS vs WT_IS	ENST00000248244	TICAM1
Hepatitis C	APTX_KO_NS vs APTX_KO_IS	ENST00000379868	DDX58 (RIG-1)	WT_NS vs WT_IS	ENST00000248244	TICAM1
Leishmaniasis	APTX_KO_NS vs APTX_KO_IS	ENST00000355622	TLR4	WT_NS vs WT_IS	ENST00000329421	MARCKSL1
Protein processing in endoplasmic reticulum	APTX_KO_NS vs APTX_KO_IS	ENST00000379484	MBTPS2	WT_NS vs WT_IS	ENST00000374492	EDEM2
Toll-like receptor signaling pathway	APTX0 NS vs APTX0 IS	ENST00000355622	TLR4	WT_NS vs WT_IS	ENST00000248244	TICAM1

1.3 **Supplementary Figure 1:** KEGG pathway plots visualizing the effects of APTX for cGAS-STING-related genes

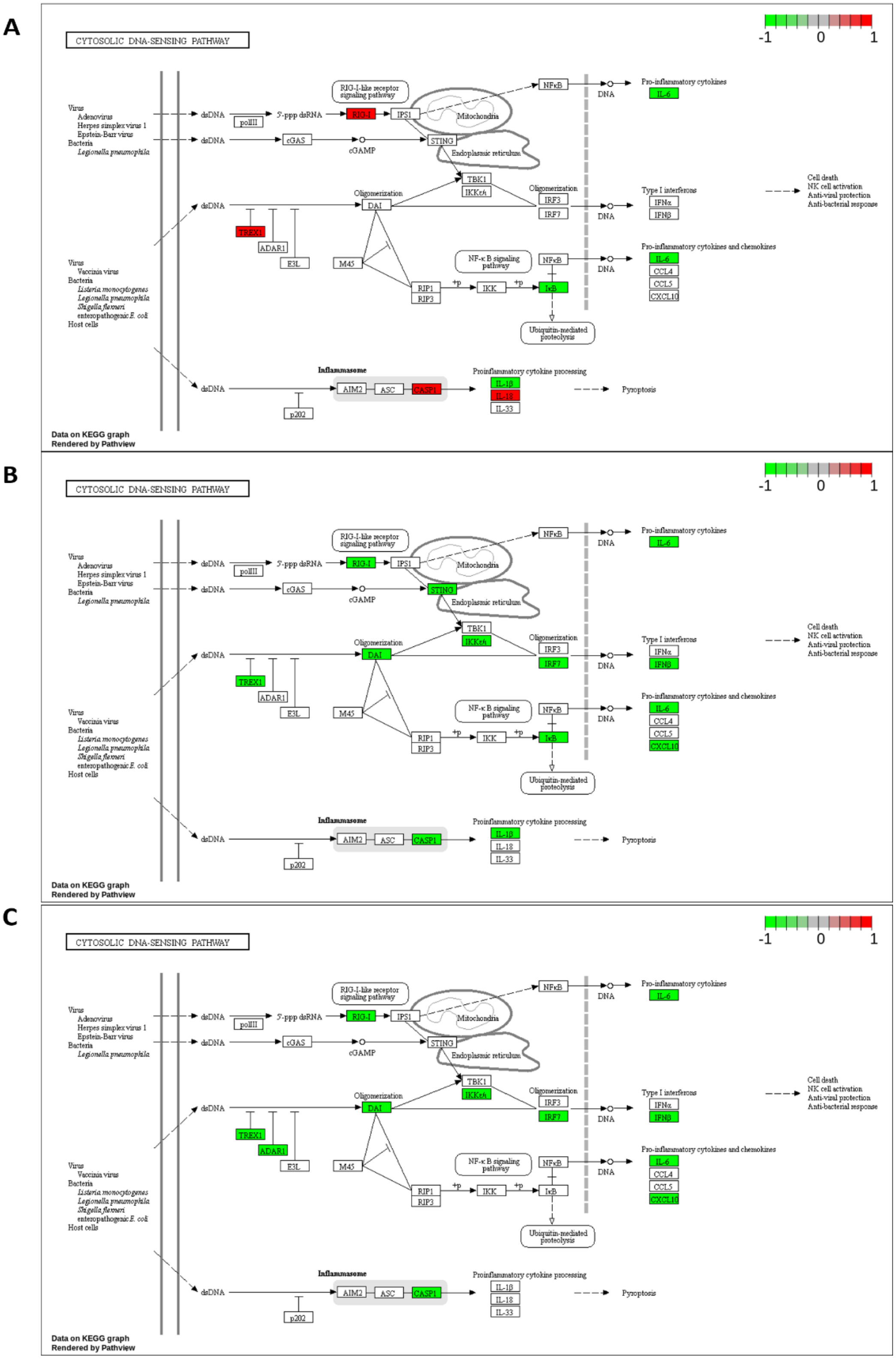

A) Rendered KEGG pathway plots for genes in the cGAS pathway significantly (q < 0.05) DE in APTX WT vs KO cell lines with no stimulation (NS visualised using a WT NS expression level

interpretation). B) cGAS pathway genes significantly (q < 0.05) DE in WT cells when adding DNA stimulation visualised using a WT and C) visualised using a KO. Where red is significantly higher expression in the first condition than the second and green is lower expression in the first condition. NS expression level interpretation.

1.4 **Supplementary Figure 2:** NF-κB is not affected by APTX-KO

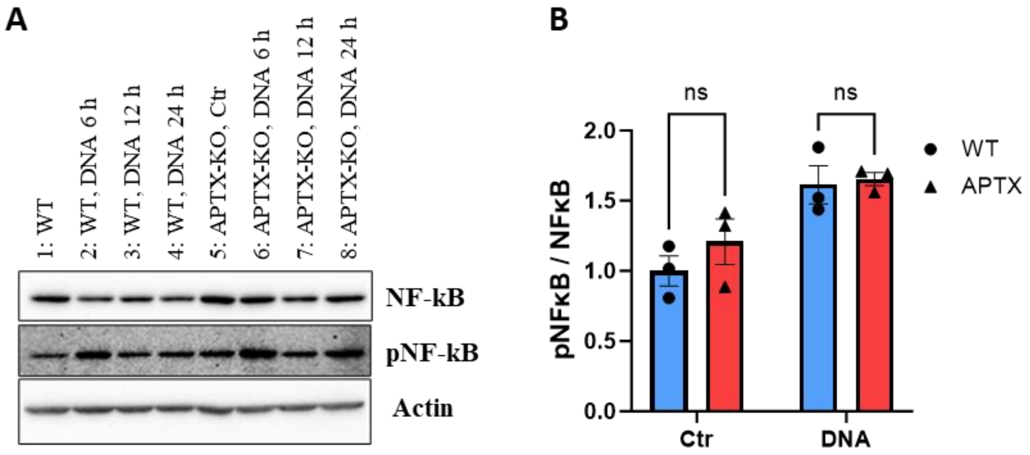

A) Western blot analysis of WT and APTX-KO cells treated with dsDNA for 6, 12 or 24h blotted for NF-κB, quantified for 12h DNA treatment in B.
